# Industrial Alkaline Electrolyzers Enabled by Interface‐Engineered Cobalt Oxide Electrodes for High‐Efficiency Water Splitting

**DOI:** 10.1002/advs.202508013

**Published:** 2025-06-23

**Authors:** Cheng Li, Xuyu Luo, Ying Wang, Mingze Zhu, Dan Li, Shiying Guo, Wei Wang, Xiaoyong Xu

**Affiliations:** ^1^ School of Physical Science and Technology & Interdisciplinary Research Center Yangzhou University Yangzhou Jiangsu 225002 China; ^2^ Jiuchang New Energy Technology Co., LTD Yangzhou 225001 China; ^3^ Jiangsu Trina Green Hydrogen Technology Co., LTD Changzhou 231021 China; ^4^ Department of Physics and Electronics School of Mathematics and Physics Beijing University of Chemical Technology Beijing 100029 China

**Keywords:** alkaline water electrolyzer, cobalt oxide electrode, high current density, hydrogen evolution reaction, interface engineering

## Abstract

Alkaline (ALK) electrolysis is an important means to generate green hydrogen from water splitting, and its technical advance hinges critically on the breakthrough of catalytic electrodes capable of high current densities at low overpotentials. Here, an efficient and robust hydrogen‐evolving electrode is developed, composed of cobalt oxide (Co_3_O_4_) nanosheet catalyst with a metal cobalt transition interface on the current‐collecting nickel wire mesh substrate. This Co_3_O_4_ electrode affords a unique charge avalanche effect at the metal–semiconductor interface to concentrate electron release and thus enables high‐current‐density hydrogen evolving at 1000 mA cm^−2^ with only 207 mV overpotential, far outperforming commercial Raney nickel electrode that commonly delivers current densities below 500 mA cm^−2^ at 300–500 mV overpotentials. An industrial cell‐stack electrolyzer utilizing Co_3_O_4_ electrodes achieves a consistent current density of 1000 mA cm^−2^ at a 2.0 V cell voltage, surpassing the operational current density of commercial ALK systems by two to five times (200–500 mA cm^−2^). A significant enhancement in current output and a minimal deactivation rate of only 0.056 mV h^−1^ demonstrate the potential of the Co_3_O_4_ electrode to replace commercial Raney nickel electrode, thereby substantially improving the hydrogen production efficiency of ALK electrolyzers.

## Graphical abstract

Alkaline electrolysis is an important means to generate green hydrogen from water splitting, and its technical advance hinges critically on the breakthrough of catalytic electrodes capable of high current densities at low overpotentials. Here cobalt oxide electrodes engineered with metal cobalt transition layers are developed to assemble an industrial electrolyzer, significantly improving alkaline water electrolysis with high current densities.

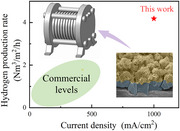

## 1 Introduction

Water electrolysis, a promising method for converting renewable electricity into storable and versatile hydrogen fuels, has garnered significant attention from both academia and industry.^[^ ^1^, ^2^, ^3^ ^]^ Among established electrolyzer architectures, including alkaline (ALK), anion exchange membrane (AEM) and proton exchange membrane (PEM) systems, ALK technology dominates >80% of industrial installations due to its operational robustness and capital cost advantages.^[^ ^4^, ^5^, ^6^ ^]^ This predominance stems from ALK's unique compatibility with earth‐abundant transition metal catalysts,^[^ ^7^, ^8^, ^9^, ^10^ ^]^ circumventing PEM's reliance on precious platinum‐group metals. Nevertheless, ALK systems suffer from inherent limitations: their operational current densities stagnate at 200–500 mA cm^−2^ under 1.8–2.4 V cell voltages, translating to oversized reactor footprints and compromised hydrogen costs that hinder gigawatt‐scale implementation.^[^ ^11^, ^12^ ^]^ As electrode overpotentials account for ≈65% of total energy consumption in ALK stacks,^[^ ^13^, ^14^, ^15^, ^16^ ^]^ developing advanced catalytic electrodes capable of sustaining high current densities beyond 1 A cm^−2^ below 300 mV overpotentials represents the critical path toward economically viable green hydrogen.

Recent advances in transition metal‐based catalysts for alkaline hydrogen evolution reaction (HER), including alloys,^[^ ^17^, ^18^ ^]^ oxides,^[^ ^19^ ^]^ carbides,^[^ ^20^, ^21^ ^]^ sulfides,^[^ ^22^, ^23^, ^24^ ^]^ and phosphides,^[^ ^25^, ^26^ ^]^ have primarily focused on intrinsic activity enhancement at laboratory‐scale current densities (<500 mA cm^−2^). However, industrial implementation demands rigorous evaluation under practical conditions where high‐current operation (>500 mA cm^−2^) exacerbates bubble shielding, catalyst detachment, and charge transfer limitation. Currently, commercial ALK electrolyzers still employ Raney nickel electrodes requiring 300–500 mV overpotentials to achieve merely 300–500 mA cm^−2^,^[^ ^27^ ^]^ a performance plateau unchanged for decades. This technological stagnation suggests an urgent need for high‐performance HER electrodes that can increase current densities without escalating overpotentials. However, the design paradigms to simultaneously optimize electron transport networks, active site exposure and interfacial stability under large current loads pose a formidable challenge.

Herein, we address this multi‐scale challenge in an interface‐engineered Co_3_O_4_ nanoarray electrode fabricated via a scalable electroplating strategy. Our design establishes a hierarchical sandwich architecture consisting of Co_3_O_4_ nanosheets with a metal Co interlayer on the nickel wire mesh (NWM), where the in situ coherent metal/oxide interface induces a quantum‐confined charge avalanche effect. This Co_3_O_4_ electrode requires an overpotential of only 207 mV to achieve a high current density of 1000 mA cm⁻^2^, remarkably surpassing the commercial Raney nickel (200–500 mA cm⁻^2^). Industrial validation in a 10‐cell‐stack electrolyzer demonstrates stable 1000 h operation at 1 A cm⁻^2^ under 2.0 V per cell, with a hydrogen production rate of 4.18 Nm^3^ m⁻^2^ h⁻¹, while achieving ampere‐level current densities previously attainable only in PEM systems. In addition, compared to energy‐intensive thermal spraying technique for fabricating Raney nickel electrodes, our room‐temperature electroplating technique reduces manufacturing costs and eliminates emissions.

## 2 Results and Discussion

### 2.1 Morphology and Structure

**Figure 1:**
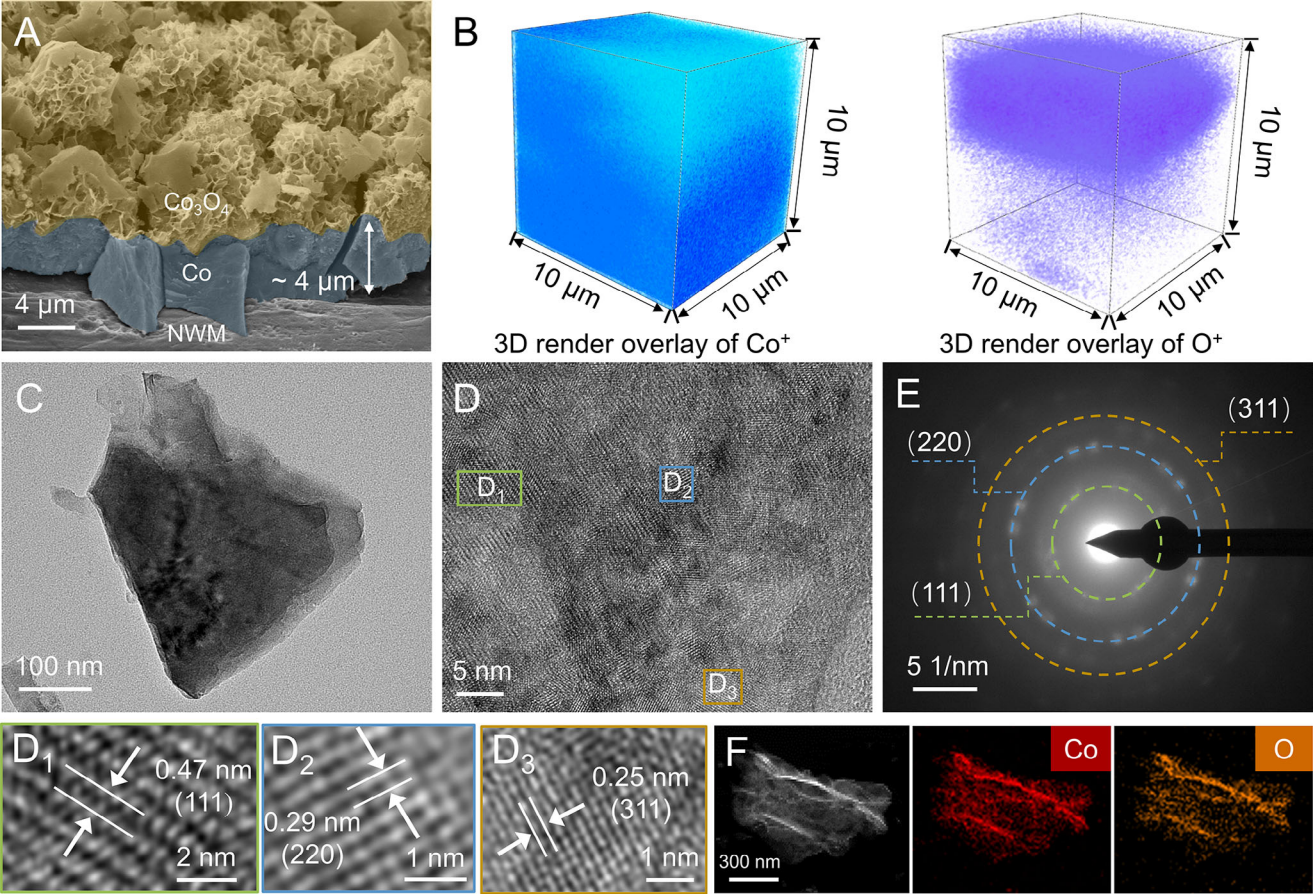
Structural characterization. A) SEM image of Co_3_O_4_ electrode. B) 3D renders of Co and O elements distributed in the TOF‐SIMS sputtered volume for Co_3_O_4_ electrode. C) TEM image of Co_3_O_4_ nanosheets. D) HRTEM image and discernible lattice analysis (D1‐D3) of Co_3_O_4_ nanosheets. E) SAED pattern of Co_3_O_4_ nanosheets. F) EDS mapping images for Co and O elements in Co_3_O_4_ nanosheets.

We developed an electroplating technique for in situ growth of Co_3_O_4_ nanosheets on the NWM substrate with interface control (see the Supporting Information for synthetic details). This synthetic approach is straightforward, efficient, and scalable for mass production. Scanning electron microscopy (SEM) images illustrate the dense growth of microflowers composed of nanosheets on the NWM skeleton (**Figure** **1**A; Figure S1, Supporting Information). A vertically hierarchical sandwich structure is evident, with an outer layer of microflowers attached to a ≈4 um thick interlayer. The porous and rough surface enables the capillary force favorable to the electrolyte penetration and gas release.^[^ ^28^ ^]^ The intimate interface ensures the mechanical adhesion of nanostructured catalyst outside, as verified by the ultrasonic destruction tests (Figure S2 and Table S1, Supporting Information), superior to the adhesion of thermal‐sprayed Raney nickel (R‐Ni) on NWM. The spatial distribution of component elements was analyzed by the time of flight secondary ion mass spectrometry (TOF‐SIMS). As shown in the 3D renders in sputtered volume (Figure 1B), Co element is dispersed throughout the volume while O element is concentrated in the upper space of 6–8 µm, which matches well with the Co_3_O_4_ layer in the SEM image (Figure 1A). Energy dispersive spectroscopy (EDS) elemental line scans (Figure S3, Supporting Information) also indicate that the interlayer primarily consists of Co, while the outer layer contains both Co and O elements. These structural features suggest the electroplating process involving two chemical stages: 1) the Co^2+^‐reduced deposition to form a Co seed layer at high Co^2+^‐ion concentration in the initial stage, followed by 2) the nucleation of Co_3_O_4_ at low Co^2+^‐ion concentration in the later stage. To confirm this process, the samples obtained at different electroplating times were observed by the SEM (Figure S4, Supporting Information). As expected, Co_3_O_4_ nanosheets do not emerge until 10 min, while they develop with uniform coverage at 15 min and then agglomerate at 20 min. Further, electroplating experiments with varying Co^2+^‐ion dosages, all conducted for 15 min, demonstrate that Co_3_O_4_ nanosheets form at a low Co^2+^‐ion concentration of 5 mm, metal Co blocks form at a high concentration of 9 mm, and a unique sandwich structure with Co/Co_3_O_4_ stacked layers on NWM only forms at a moderate Co^2+^‐ion concentration of 7 mm (Figure S5, Supporting Information). This observation confirms that the deposition mechanism is contingent upon the Co^2+^‐ion concentration. Through a comparison of HER performance (Figure S6, Supporting Information), an electroplating for 15 min at a Co^2+^‐ion concentration of 7 mm is optimal for fabricating such a metal Co interface‐engineered Co_3_O_4_ electrode with maximum HER performance.

The nanosheets, stripped using intense ultrasound, were further analyzed via the transmission electron microscopy (TEM), revealing the 2D lamellar structure (Figure 1C). The high‐resolution TEM (HRTEM) displays lattice fringes dispersed across various regions of the nanosheet (Figure 1D), corresponding to the (111), (220), and (311) phases of spinel Co_3_O_4_ (Figures D1, D2, and D3), as confirmed by the selected area electron diffraction (SAED) pattern (Figure 1E). The EDS spectrum reveals the existence of predominant Co and O elements (Figure S7, Supporting Information), which are uniformly distributed within the nanosheets, as shown in the elemental mapping images (Figure 1F). The X‐ray diffraction (XRD) spectrum shows no discernible peaks, while the Raman spectrum exhibits weak peaks characteristic of spinel Co_3_O_4_ (Figure S8, Supporting Information), with vibration modes of *A*_1g_, *F*_2g_, and *E*_g_ attributed to Co^2+^/^3+^‐O bonds in tetrahedral and octahedral units.^[^ ^29^, ^30^ ^]^ Consequently, the quasi‐crystalline nature of Co_3_O_4_ component is determined, consistent with HRTEM and SAED analyses. These results elucidate a sandwich‐structured electrode produced through a facile electroplating technique, wherein low‐crystallinity Co_3_O_4_ nanosheets grown on the Co seed layer serve as the outer catalyst.

### 2.2 Chemical Composition and State

**Figure 2:**
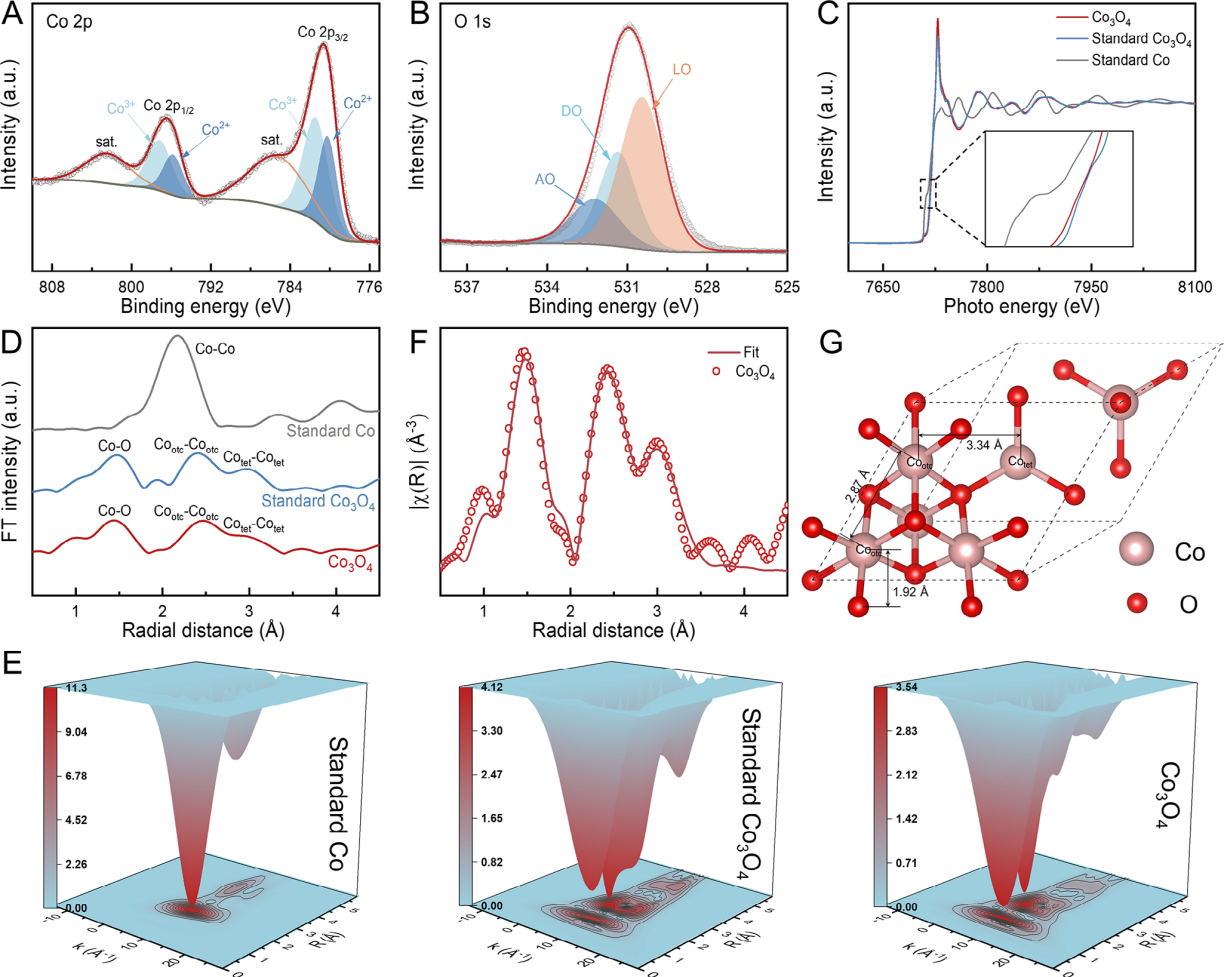
Chemical state characterization. A,B) High‐resolution XPS spectra on Co‐2p (A) and O‐1s (B) cores of Co_3_O_4_ electrode. C) Co K‐edge XANES spectra of Co_3_O_4_ electrode, with standard Co_3_O_4_ and Co references. D) FT‐EXAFS spectra at the Co K‐edge of Co_3_O_4_ electrode and standard references. E) Co K‐edge WT‐EXAFS of Co_3_O_4_ electrode and standard references. F) FT‐EXAFS spectrum fitting of Co_3_O_4_ electrode at Co K‐edge. G) Atomic model of spinel Co_3_O_4_ structure.

The X‐ray photoelectron spectroscopy (XPS) was employed to explore the chemical composition and state of the as‐prepared Co_3_O_4_ electrode. The XPS survey spectrum (Figure S9, Supporting Information) identifies the composition of O and Co elements, with trace Ni originating from the NWM substrate. In **Figure** **2**A, the XPS spectrum at Co‐2p core shows spin‐orbit peaks at 780.7 and 796.5 eV for 2p_3/2_ and 2p_1/2_, respectively, which are deconvoluted into the constituent peaks representing Co^2+^ and Co^3+^ states of spinel Co_3_O_4_.^[^ ^31^ ^]^ In addition, after sputtering with Ar ions, a new dominant peak at 778.3 eV emerges in Co‐2p profile (Figure S10, Supporting Information), which is attributed to zero‐valent Co° component, further confirming the existence of interbedded metal Co species. The O‐1s peak contains three deconvoluted peaks at 530.4, 531.3, and 532.2 eV (Figure 2B), ascribed to the lattice oxygen (LO), defective oxygen (DO) and adsorbed oxygen (AO) in ‐OH or H_2_O groups on the surface.^[^ ^32^, ^33^ ^]^ The X‐ray absorption spectroscopy (XAS) was then conducted for further analysis of chemical states. The Co K‐edge X‐ray absorption near‐edge structure (XANES) spectra (Figure 2C) confirm the valence state of Co cations in alignment with the standard spinel Co_3_O_4_ reference, distinct from the Co foil reference. The Fourier transform‐extended X‐ray absorption fine structure (FT‐EXAFS) spectra at Co K‐edge (Figure 2D) distinguish three interatomic distances that are characteristic of Co─O, octahedral Co_oct_‐Co_oct_ and tetrahedral Co_tet_‐Co_tet_ in the Co_3_O_4_ phase.^[^ ^34^, ^35^ ^]^ The wavelet transform‐EXAFS (WT‐EXAFS) analysis at Co K‐edge shows three characteristic regions with one Co─O and two Co─Co shell scatterings (Figure 2E), in good agreement with the standard Co_3_O_4_ reference. In addition, the fitting results of Co K‐edge FT‐EXAFS spectra (Figure 2F; Figure S11, Supporting Information) determine the coordination numbers (CN) of Co‐O, Co_oct_‐Co_oct_ and Co_tet_–Co_tet_ to be 3.38, 6.55 and 3.67 (Table S2, Supporting Information), respectively, which closely resembling to the theoretical values in the spinel Co_3_O_4_ structure (Figure 2G). These findings definitively confirm that the nanosheet catalyst loaded through the electroplating process is indeed spinel Co_3_O_4_.

### 2.3 High‐Current‐Density HER Performance

**Figure 3:**
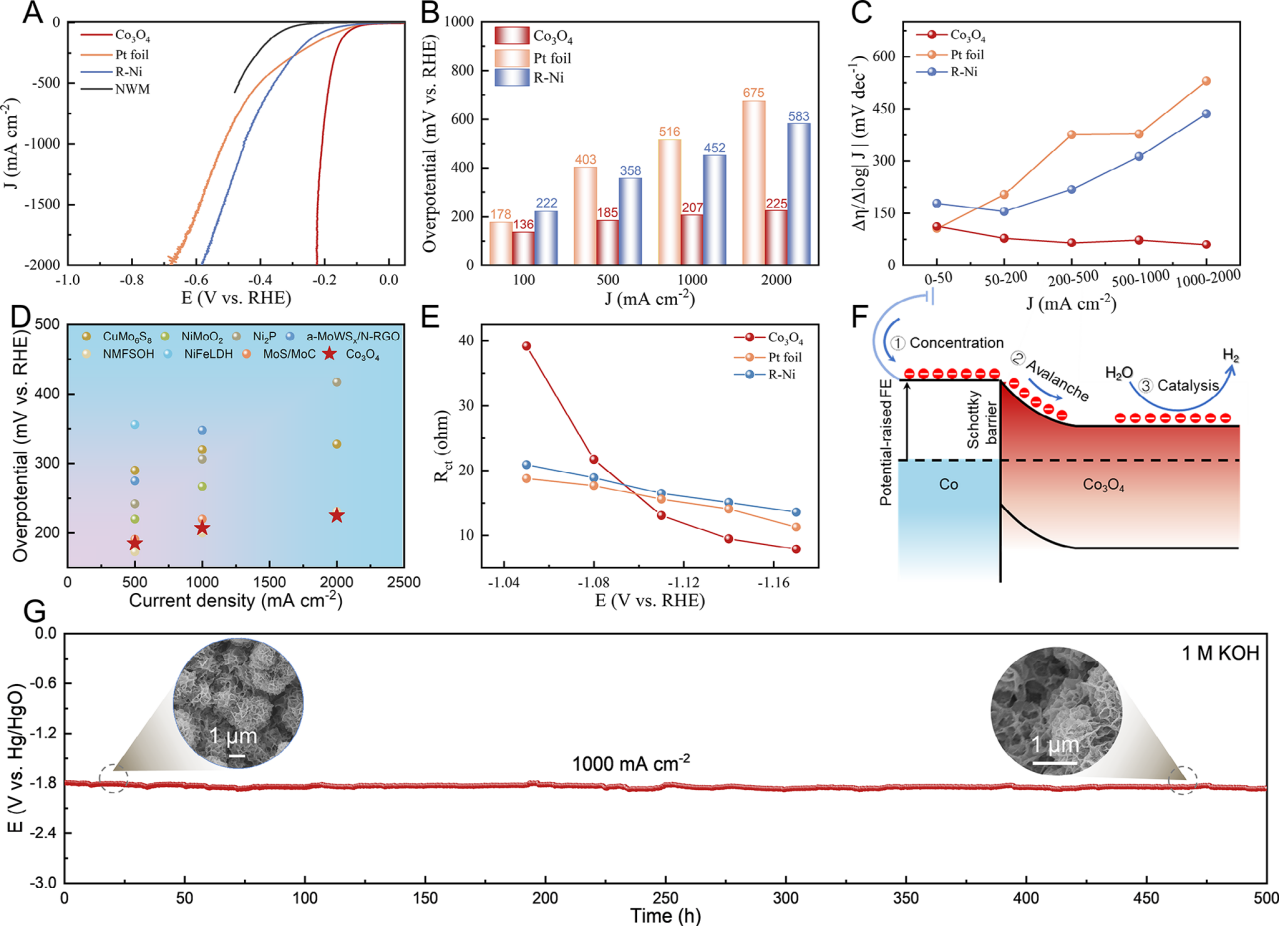
HER performance evaluation. A) Polarization curves of Co_3_O_4_, bare NWM, commercial R─Ni and Pt foil measured in 1 m KOH with 85% iR correction. B) Comparison of overpotentials at typical current densities (100, 500, 1000, and 2000 mA cm^−2^) for Co_3_O_4_, R─Ni and Pt electrodes. C) Ratios of Δη/Δlog|j| at various current density ranges of Co_3_O_4_, R─Ni and Pt electrodes. D) Comparison of overpotentials at typical current densities (500, 1000, and 2000 mA cm^−2^) for Co_3_O_4_ with state‐of‐the‐art catalysts reported in literatures (Table S3, Supporting Information). E) *R*_ct_ value as a function of external potential for Co_3_O_4_, R‐Ni and Pt electrodes. F) Schematic illustration of charge concentration and avalanche into catalysis at M–S interface. G) CP stability test at a constant current density of 1000 mA cm^−2^ for Co_3_O_4_ electrode measured in 1 m KOH, with SEM images before and after stability test (insets).

The HER performance of Co_3_O_4_ was measured via the line sweep voltammetry (LSV) at room temperature using the three‐electrode mode in 1 m KOH electrolyte, with the bare NWM, commercial R─Ni and Pt foil as three references. The polarization curves were calibrated with 85% *iR* correction for fair comparison (**Figure** **3**A), and the raw data before the correction were shown in Figure S12 (Supporting Information). Notably, Co_3_O_4_ has much higher HER activity compared to R─Ni and Pt, especially under high current densities. The overpotentials required to reach 1000 and 2000 mA cm^−2^ are only 207 and 225 mV for Co_3_O_4_ electrode (Figure 3B), much lower than those for R─Ni (452 and 583 mV) and Pt foil (516 and 675 mV). Further, the low ratios of Δη/Δlog|j| are consistently less than 80 mV dec^−1^ across various current density ranges for Co_3_O_4_ electrode in contrast with other electrodes (Figure 3C), demonstrating that it enables fast mass transfer conducive to high‐current‐density catalysis.^[^ ^36^ ^]^ A comparison on overpotentials with state‐of‐the‐art catalysts in Figure 3D (Table S3, Supporting Information) underscores the superior performance of Co_3_O_4_ electrode at high current densities over 500 mA cm^−2^. Controlled experiments involving variations in electroplating time and cobalt dosage were conducted to optimize the HER performance (Figure S6, Supporting Information), resulting in the synthesis of a sandwich‐structured sample with the highest HER activity achieved through electroplating with 7 mmol of cobalt ions for 15 min. This indicates the critical importance of the sandwich structure for enhancing HER performance.

### 2.4 High‐Current‐Density HER Mechanism

To understand the HER performance at high current densities, the charge transfer kinetics, active‐site number and intrinsic activity were evaluated for Co_3_O_4_, R‐Ni and Pt foil electrodes.^[^ ^37^, ^38^, ^39^ ^]^ We extracted the interfacial charge transfer resistance (*R*_ct_) from the electrochemical impedance spectra (EIS, Figure S13, Supporting Information) and depicted its dependence on the applied potential in Figure 3E. At a low potential of −1.05 V versus Hg/HgO, Co_3_O_4_ shows much higher *R*_ct_ than R─Ni and Pt, due to its semiconducting nature. Notably, a significant reduction in *R*_ct_ is evident in Co_3_O_4_ with increasing potentials, while Pt and R─Ni exhibit a gradual decrease contrasting with the former. Interestingly, when the potential increased to −1.17 V versus Hg/HgO, Co_3_O_4_ instead exhibits the smallest *R*_ct_ of 4.8 Ω, with respect to R─Ni (10.6 Ω) and Pt (8.1 Ω). Consistently, Bode phase plots of dynamic EIS spectra (Figure S14, Supporting Information) also reveals the external potential‐regulated charge transfer kinetics in Co_3_O_4_ different from R─Ni and Pt. In addition, the cyclic voltammetry (CV) was measured without Faraday current to determine the double layer capacitance (*C*_dl_) (Figure S15, Supporting Information).^[^ ^40^, ^41^, ^42^ ^]^ Co_3_O_4_ exhibits the higher *C*_dl_ of 53.8 mF cm^−2^ than R─Ni (14.3 mF cm^−2^) and Pt (34.2 mF cm^−2^), indicating its larger electrochemical surface area (ECSA) with more accessible active sites. The ECSA‐normalized current densities (*J*_ECSA_) and turnover frequencies (TOF) were calculated and present in Figure S16 (Supporting Information). At low current densities, Pt foil exhibits superior intrinsic activity; however, at high current densities, the TOF of Pt foil is surpassed by porous R─Ni and Co_3_O_4_ electrodes, indicating that factors related to mass/charge transport become more critical to catalytic kinetics.

The dramatic acceleration of charge transfer kinetics for Co_3_O_4_ upon applied potentials can be attributed to the distinctive metal–semiconductor (M–S) interface within the sandwich structure. As schematically illustrated in Figure 3F, the Schottky barrier is formed at the M–S interface due to different work functions on each side (Figure S17, Supporting Information). This results in electron concentration on the metal Co side under negative potentials, accompanied by an elevation of the Fermi level (FL). Upon reaching a threshold potential that overcomes the Schottky barrier, the accumulated electrons are collectively released toward the Co_3_O_4_ side, triggering the charge avalanche effect favorable to the intensive HER at high current densities. Note that semiconductor‐type catalysts have historically been overlooked in electrocatalysis due to their poor conductivity. The interfacial charge avalanche mechanism establishes a new design paradigm for semiconductor‐based electrocatalysts.

Besides, the ordered nanostructure of Co_3_O_4_ catalyst accelerates mass transfer, also crucial for the high‐current‐density catalysis. Brunauer–Emmett–Teller (BET) analysis (Figure S18, Supporting Information) reveals that Co_3_O_4_ has high specific surface area and porosity, indicating its geometric advantage over the randomly accumulated R─Ni particles on the NWM substrate in terms of active‐site exposure, reactant accessibility, and bubble release. Further, dynamic droplet wetting tests demonstrate superior surface wettability of Co_3_O_4_ compared to R─Ni and Pt (Figure S19, Supporting Information), benefitting from the viable capillary force in the ordered nanostructure. Moreover, higher hydrophilicity means more gas‐phobic on the surface,^[^ ^43^, ^44^ ^]^ which would reduce the interfacial bubble adhesion to accelerate the gas release. Thus, the improved mass transfer of aqueous reactants and gaseous products in Co_3_O_4_ electrode also contributes to its favorable HER performance at high current densities.

### 2.5 High‐Current‐Density HER Stability

The stability at high current densities is an important criterion to determine the feasibility of an electrode in practical applications. Chronopotentiometry (CP) test for Co_3_O_4_ electrode demonstrates a stable operation at 1000 mA cm^−2^ over a period of 500 h, with a negligible activity decay rate of only 0.06 mV h^−1^ (Figure 3G). In contrast, the CP test for R─Ni electrode at 1000 mA cm^−2^ shows a marked decline in activity within 120 h (Figure S20, Supporting Information). Additionally, Faraday efficiencies were evaluated to be nearly 100% by quantifying hydrogen production through gas chromatography (Figure S21, Supporting Information), indicating high HER selectivity of Co_3_O_4_ without any side reactions. Consistent polarization curves observed before and after the stability assessment (Figure S22, Supporting Information) further validate the excellent durability of Co_3_O_4_ electrode under high‐current‐density HER operation. Subsequent structural analyses of Co_3_O_4_ electrode used after the stability test, utilizing XRD, SEM, TEM, and XPS techniques (Figure S23, Supporting Information), reveal no significant alterations in morphology, texture, composition, or valence states before and after the stability test. The results collectively suggest the potential suitability of Co_3_O_4_ electrode for use in practical ALK electrolyzers.

### 2.6 Cost‐Effectiveness of Electroplating Technique

**Figure 4:**
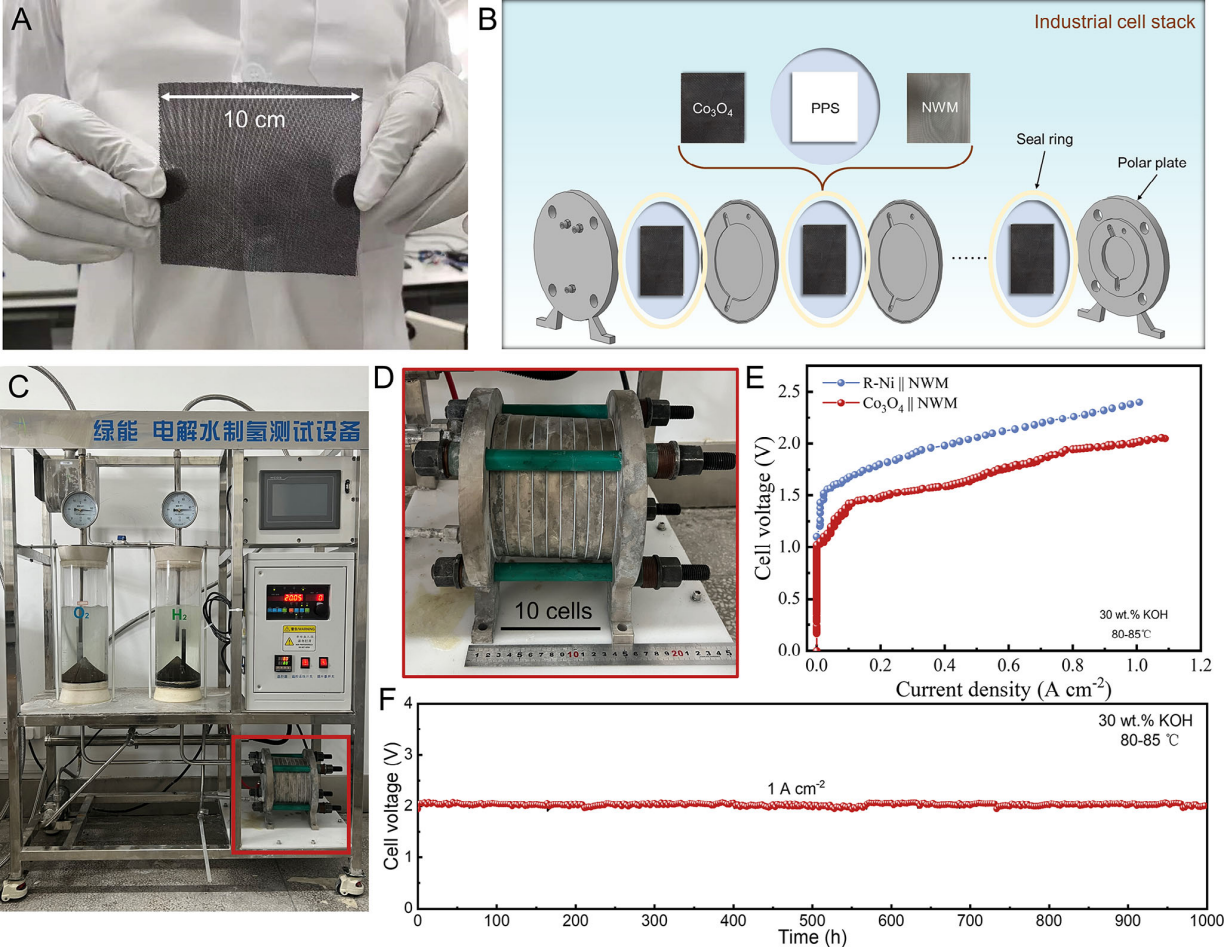
Industrial cell‐stack system performance evaluation. A) Photograph of a scalable Co_3_O_4_ electrode. B) Schematic of the cell‐stack Co_3_O_4_‖NWM electrolyzer. C,D) Photographs of an industrial electrolysis system (C) and a cell‐stack electrolyzer (D) circled by red box in (C). E) LSV curves of cell‐stack Co_3_O_4_‖NWM and R‐Ni‖NWM electrolyzers measured under industrial conditions (6 m KOH at 80–85 °C). F) Time‐dependent cell voltage of Co_3_O_4_‖NWM electrolyzer operating at 1000 mA cm^−2^ under industrial conditions (6 m KOH at 80–85 °C).

The electroplating method to prepare Co_3_O_4_ nanoarrays on the NWM substrate can be scaled up for industrial production. The choice of nickel mesh rather than cobalt mesh as the substrate is primarily due to the lower cost of nickel. A photograph in **Figure** **4**A displays a typical Co_3_O_4_ electrode of 100 cm^2^ in size, and SEM images at different regions (Figure S24, Supporting Information) manifest the uniform coverage of catalysts across the electrode, with a loading of 10 mg cm^−1^ (Table S1, Supporting Information). The price of Co_3_O_4_ electrode was estimated to be ≈45.0 US$ m^−2^ based on the raw material and labor costs (Table S4, Supporting Information), which is much lower than that of commercial R‐Ni (≈72.4 US$ m^−2^). In contrast to the energy‐intensive thermal spraying technique used for R─Ni electrode preparation, electroplating method offers a more energy‐efficient and eco‐friendly alternative suitable for large‐scale production. Notably, electroplating allows for the construction of functional architectures to enhance charge/mass transfer, favorable to high‐current‐density catalysis. Nevertheless, the utilization of electroplated electrodes in industrial‐grade ALK electrolyzers remains unexplored to date.

### 2.7 Industrial‐Grade Electrolyzer Performance

We assembled the Co_3_O_4_‖NWM electrolyzer with ten single cells using Co_3_O_4_ cathodes and NWM anodes, with the same area of 9 cm^2^. Figure 4B shows the cell stack configuration, including Co_3_O_4_ cathodes and NWM anodes, polyphenylene sulphide (PPS) membranes, seal rings and polar plates. For comparison, an industrially utilized R‐Ni‖NWM electrolyzer was also assembled using commercial R─Ni cathodes and NWM anodes. An industrial electrolysis setup (Figure 4C) was customized with the cell‐stack electrolyzer (Figure 4D) to evaluate practical water splitting performance under industrial conditions (30% KOH and 80–85 °C). The LSV curves in Figure 4E display the performance contrast between the two electrolyzers. The current densities of the Co_3_O_4_‖NWM electrolyzer are consistently higher than that of the R‐Ni‖NWM across all voltages, indicating a valuable ability to increase hydrogen output and reduce facility cost. The Co_3_O_4_‖NWM electrolyzer reaches current densities of 500 and 1000 mA cm^2^ at cell voltages of 1.77 and 2.0 V, notably outperforming the R‐Ni‖NWM electrolyzer that requires cell voltages more than 2.06 V to attain current densities above 500 mA cm^−2^.

Further, the Co_3_O_4_‖NWM electrolyzer demonstrates long‐term CP stability at 1000 mA cm^−2^ for over 1000 h (Figure 4F), maintaining the cell voltage at around 2.0 V, with a minimal decay rate of 0.056 mV h^−1^. Video S1 (Supporting Information) records the consistent voltage and current in an industrial electrolysis system, along with the vigorous bubbling of hydrogen and oxygen. This stable operation at 1000 mA cm^−2^ enables high‐throughput hydrogen production at a rate of 4.18 Nm^3^ m^−2^ h^−1^, comparable to precious metal‐base PEM electrolyzers.^[^ ^45^ ^]^ This advancement of increasing operational current density significantly cuts down on the requirement to enlarge equipment volume for large‐scale hydrogen production. Consequently, the estimated cost of hydrogen production using the Co_3_O_4_‖NWM electrolyzer is ≈1.15 US$ kg^−1^, below the 2026 target of 2.0 US$ kg^−1^ from U.S. Energy Department.^[^ ^46^ ^]^ In sum, the reliable performance of Co_3_O_4_ electrode in an industrial‐grade electrolyzer sets the stage for its application to advance ALK technology.

## 3 Discussion

In summary, we report the performance of a unique Co_3_O_4_ catalyst electrode prepared by electroplating process and its application in an industrial electrolyzer, thus demonstrating the viable substitution for Raney nickel electrode to update alkaline water electrolyzers. The Co_3_O_4_ electrode exhibits the sustainable HER at a high current density of 1000 mA cm^−2^ with an overpotential as low as 207 mV. A cell‐stack electrolyzer assembled with Co_3_O_4_ electrodes performs steady water electrolysis of 1000 mA cm^−2^ at a cell voltage of 2.0 V under industrial conditions, significantly increasing hydrogen yield compared to the traditional Raney nickel‐based electrolyzer with low current densities less than 500 mA cm^−2^. Besides, electroplating synthesis of Co_3_O_4_ electrode with low cost and no pollution is amenable to large‐scale implementation. These results identify a high‐performance Co_3_O_4_ electrode for alkaline HER and suggest its adoption to upgrade alkaline water electrolyzers in industry.

## Conflict of Interest

The authors declare no conflict of interest.

## Author Contributions

C.L., X.L., and Y.W. contributed equally to this work. X.X. conceived the project. C.L. and X.L. designed the experiments and performed the characterizations and tests. Y.W. and S.G. performed the DFT calculations. M.Z. and D.L. designed and assembled the electrolysis system. W.W. and X.X. analyzed the data and wrote the manuscript. All authors revised and approved the manuscript.

## Supporting information



Supporting Information

Supplemental Video 1

## Data Availability

The data that support the findings of this study are available from the corresponding author upon reasonable request.
