# Supplementary material for: Industrial Alkaline Electrolyzers Enabled by Interface‐Engineered Cobalt Oxide Electrodes for High‐Efficiency Water Splitting
Source: Adv Sci (Weinh). 2025 Jun 23;12(35):e08013. doi: 10.1002/advs.202508013 (PMC12462948; doi:10.1002/advs.202508013)
Supplement: Supplementary file 1 — Supporting Information [file ADVS-12-e08013-s001.pdf]

ADVANCED SCIENCE
Open Access

Supporting Information

for *Adv. Sci.*, DOI 10.1002/advs.202508013

Industrial Alkaline Electrolyzers Enabled by Interface-Engineered Cobalt Oxide Electrodes for High-Efficiency Water Splitting

Cheng Li, Xuyu Luo, Ying Wang, Mingze Zhu, Dan Li, Shiying Guo, Wei Wang and Xiaoyong Xu**

Supporting Information

Industrial Alkaline Electrolyzers Enabled by Interface-Engineered Cobalt Oxide Electrodes for High-Efficiency Water Splitting

Cheng Li,[#] *Xuyu Luo,*[#] *Ying Wang,*[#] *Mingze Zhu, Dan Li, Shiying Guo, Wei Wang,* and Xiaoyong Xu**

Dr. C. Li, Dr. X. Luo, Dr. Y. Wang, Dr. S. Guo, and Prof. X. Xu

School of Physical Science and Technology & Interdisciplinary Research Center, Yangzhou University, Yangzhou, Jiangsu 225002, China.

E-mail: xxy@yzu.edu.cn

Dr. M. Zhu

Jiuchang New Energy Technology Co., LTD, Yangzhou 225001, China.

Dr. D. Li

Jiangsu Trina Green Hydrogen Technology Co., LTD, Changzhou 231021, China.

Prof. W. Wang

Department of Physics and Electronics, School of Mathematics and Physics, Beijing University of Chemical Technology, Beijing 100029, China.

E-mail: wangwei@mail.buct.edu.cn

[#]These authors contributed equally: Cheng Li, Xuyu Luo, Ying Wang

Experimental Section

Chemicals: Thiourea (CH_4N_2S), potassium hydroxide (KOH), cobalt (II) acetate tetrahydrate $(CH_3COO)_2Co\cdot4H_2O$, ammonium chloride (NH_4Cl), ethanol, and nitric acid were purchased from China National Pharmaceutical Group Chemical Reagent Co., Ltd. Nickel wire mesh (NWM) was supplied by Jiangsu Green Hydrogen Electrode Co., Ltd. NWM-supported Raney® nickel (R-Ni) electrode fabricated by thermal spraying technique was purchased from Jiangsu Leini Metal Technology Co., Ltd. Platinum (Pt) foil, carbon rod and Hg/HgO electrodes were purchased from Shanghai Chenhua Co., Ltd. Deionized water was homemade in our lab.

Synthesis of Co_3O_4 electrode: The NWM was pretreated sequentially with nitric acid, ethanol, and deionized water, with each treatment involving 5 minutes of sonication to remove organic compounds from its surface and enhance its hydrophilicity. Then, 1.74 g of $(CH_3COO)_2Co\cdot4H_2O$, 0.53 g of CH_4N_2S, and 7.5 g of NH_4Cl were dissolved in 70 ml of deionized water and thoroughly stirred for half an hour to obtain a clear reddish-purple solution. The mixture was used as an electrolyte, and two cleaned NWM slices of the same size (2 cm × 3 cm) were employed as electrodes, constructing a two-electrode electroplating system. The electroplating process was performed with a galvanostatic method, maintaining a current density of 200 mA cm^{-2}. The Co-ion dosages (5, 7 and 9 mM) and electroplating times (600, 900 and 1200 s) were regulated for sample screening to obtain the optimal HER performance.

Material Characterizations: XRD was conducted on a Bruker AXS D8 Advance X-ray diffractometer using a Cu Kα radiation source (λ = 1.5418 Å). SEM was taken with a Hitachi S-4800II instrument operated at 5.0 kV. TEM were taken on a FEI Tecnai G2F30 instrument at 300 kV. X-ray absorption spectroscopy was performed at the BL11B beamline of the Shanghai Synchrotron Radiation Facility (SSRF). TOF-SIMS

was conducted on a TOF-SIMS 5-100 instrument of Suzhou Institute of Nano-Tech and Nano-Bionics (SINTNB), with a 30-keV Bi^{3+} sputtering source. Raman spectroscopy was performed on a Renishaw inVia confocal microscope using a 63x water immersion objective (Leica Microsystems). XPS data were collected on an ESCALAB250Xi spectrometer using an Al Kα excitation source, with a standard calibration according to the C 1s peak at 284.8 eV. Porosity and BET analyses were conducted on an Autosorb-iQ3 (Quantachrome, USA) using nitrogen as the adsorptive medium. The ultrasound machine was purchased from Kunshan Hechuang Ultrasonic Instrument Co., Ltd., model KH-250DE. Gas evolution was online collected and quantified using a gas-seal cell system with a FULI 9790H chromatograph during water electrolysis at 1000 mA cm^{-2}.

Electrochemical tests: Electrochemical tests in three-electrode model were performed on a CHI114D electrochemical station in 1 M KOH electrolyte at room temperature (~ 25 ℃). Different contrast electrodes with the same effective size of 0.5 cm × 0.5 cm served as working electrodes, using a Hg/HgO and a carbon rod as the reference and counter electrodes, respectively. The electrocatalytic performance was assessed using steady LSV curves measured at a sweep rate of 5 mV s^{-1}. Unless otherwise specified, all potentials were converted against the RHE and calibrated with 85% iR compensation, based on the following standard equations:

$$\mathrm{E_{RHE}} = \mathrm{E_{Hg/HgO}} + 0.098 + 0.059 \times \mathrm{pH} \quad (1)$$

$$E_{\mathrm{reported}} = E_{\mathrm{RHE}} - 85\% iR_{\mathrm{s}} \quad (2)$$

where $E_{\mathrm{Hg/HgO}}$, E_{RHE} and E_{reported} are the measured potential vs. Hg/HgO reference, the converted potential vs. RHE and the reported potential with $85\% iR_{\mathrm{s}}$ compensation; pH value of 1 M KOH electrolyte at room temperature is measured 14;

0.098 is a standard potential value of Hg/HgO vs. RHE at room temperature; i is the current that equals to the product of current density and electrode size (0.25 cm^2); R_s denotes the series resistance that is obtained to be 3.2 Ω from EIS spectra.

Practical water electrolysis tests: The two actual electrolyzers were assembled from 10 cell stacks, with polyphenylene sulphide (PPS) membranes, seal rings and polar plates. For the Co_3O_4‖NWM electrolyzer, 3×3 cm^2 NWM-supported Co_3O_4 and bare NWM were used as cathodes and anodes, respectively. Commercial Raney Ni covered on NWM by thermal spraying were used as control cathodes, with bare NWM as anodes in the R-Ni‖NWM electrolyzer. Practical water electrolysis was conducted with a LW3030KD DC power supply under industrial conditions with flowing 30% KOH electrolyte at 80-85 °C. The LSV and CP tests with the industrial-scale large current densities were performed using a direct-current rectifier instead of an electrochemical workstation. The original LSV and CP data measured by the electrolyzers were reported without undergoing *iR* calibration.

TOF assessment: The C_{dl} was assessed by depicting the linear relationship between half of the current density difference at the central position of the cyclic voltammetry ($\Delta j/2$) and the voltammetry scan rates (v) with the equation 3:

$$C_{\mathrm{dl}} = \frac{\Delta j/2}{v} \quad (3)$$

The ECSA was calculated based on C_{dl} and standard reference (C_{s}) valued as 0.04 and 0.62 mF cm^{-2} for nanostructured Co_3O_4 and Pt foil electrodes, respectively, according to the equation 4:

$$\mathrm{ECSA} = C_{\mathrm{dl}}/C_{\mathrm{s}} \quad (4)$$

The TOF was calculated by the equation 5:

$$\mathrm{TOF} = \frac{j}{2Fn} \quad (5)$$

where j is the current density (A m^{-2}), 2 is the electron number in one HER, F refers to the Faraday's constant (96500 C/mol), n is the reaction-site mole number per square meter (mol m^{-2}), which was calculated by the equation 6:

$$n = \frac{C_{dl} \times \Delta V}{2F} \quad (6)$$

where C_{dl} is double-layer capacitance value (F m^{-2}), ΔV is the CV voltage range, F is the Faraday's constant (96500 C/mol), and 2 represents the charge-discharge cycles.

Faradaic Efficiency Calculation: The theoretical H_2-evolved amount ($M_{H_2}^{theo}$, mol) was calculated basing on the real-time current density (j, 1 A cm^{-2}) using the following equation 7, and thus the Faradaic efficiency (FE) was evaluated by the equation 8.

$$M_{H_2}^{exp} = \frac{jAt}{2eN_A} \quad (7)$$

$$FE = \frac{M_{H_2}^{exp}}{M_{H_2}^{theo}} \times 100\% \quad (8)$$

where A is the effective electrode area (0.25 cm^2), e is the charge of one electron (1.6022 × 10^{-19} C), t is the reaction time, N_A is Avogadro's number (6.022 × 10^{23}), and $M_{H_2}^{exp}$ is the experimentally measured amount of H_2 evolution.

Activity degradation rate (D_V*) calculation*: We took the average of the potential within the initial and final 10% time, defined as V_1 and V_2, in the CP test over duration time (*t*) to calculate the D_V by the following equation:

$$D_V = \frac{V_2 - V_1}{t} \quad (9)$$

Electrolyzer Performance Parameter Calculations: The volume of hydrogen per mole is 22.4×10^{-3} Nm^3 in the standard state, so unit electric quantity (Q) was calculated with the avogadro number (N_A) and electron charge (e) by the equation 9:

$$Q = \frac{2N_A e}{3600 \times 22.4 \times 10^{-3}} = 2390 \ \text{A h Nm}^{-3} \quad (10)$$

And then electricity consumption (W) was calculated at a cell voltage (V =2.0 V) by the following equation 10:

$$W = Q \times \frac{V}{1000} \quad \text{kW h Nm}^{-3} \qquad (11)$$

The price of hydrogen production (P_{H}) was estimated with a hydrogen density (ρ = 0.083 kg Nm^{-3}) in the standard state and a commercial tariff of approximately US$ 0.02 (kW h)$^{-1}$ by the equation 11:

$$P_{\mathrm{H}} = \frac{W \times 0.02}{\rho} \quad \text{US\$ kg}^{-1} \qquad (12)$$

The hydrogen production rate (R_{H}) was calculated from equation 12 with an operating current density (j = 10000 A m^{-2}).

$$R_{\mathrm{H}} = \frac{j}{Q} \ \text{Nm}^{3}\ \text{h}^{-1}\ \text{m}^{-2} \qquad (13)$$

Density Functional Theory Calculations: All density functional theory (DFT) calculations in this work were taken in the Vienna Ab-initio Simulation Package (VASP). The exchange-correction interaction was described via the Perdew-Burk-Ernzerhof function. Six layers of Co (111) were used and the top two atomic layers were relaxed during the structural optimization, and the other four metal layers were fixed in their crystal structures. The cut-off energy was 500 eV. Each system was fully relaxed with the interatomic forces less than 0.01 eV/Å and the energy difference below 10^{-5} eV. The Monkhorst-Pack k-point grid was set to 11 × 11 × 1 for Co (111) and 5 × 8 × 1 for Co_3O_4 (220).

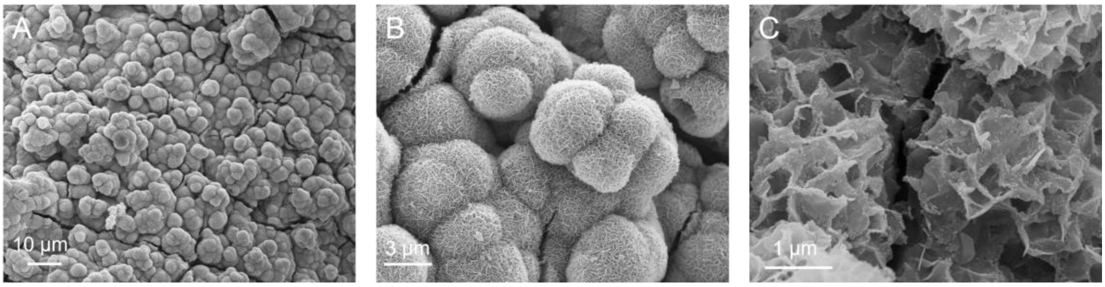

Figure S1. SEM images with different scales of Co_3O_4 electrode.

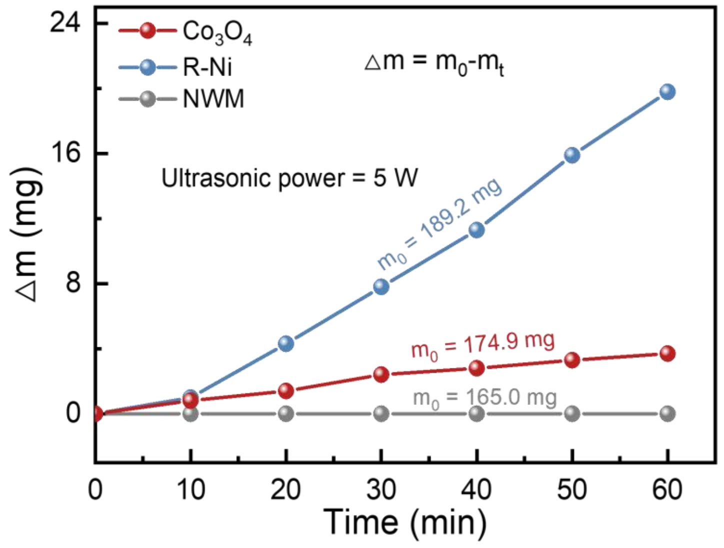

Figure S2. Time-dependent mass losses in the ultrasonic destruction tests for Co_3O_4, R-Ni, and NWM electrodes, with measured values in Table S1.

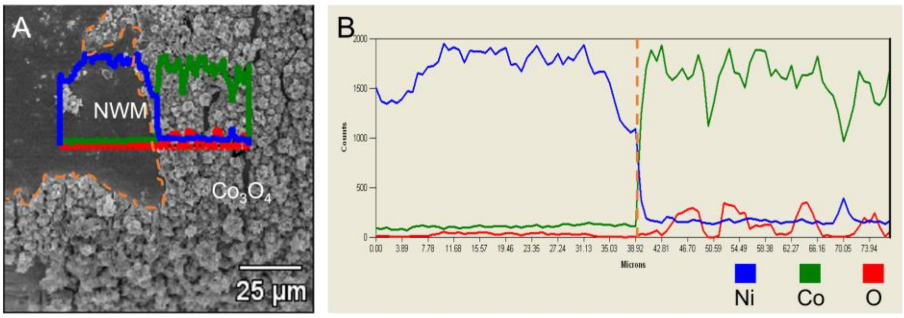

Figure S3. EDS line scans of Ni, Co, and O elements for Co_3O_4 electrode with partially detached catalysts on surface.

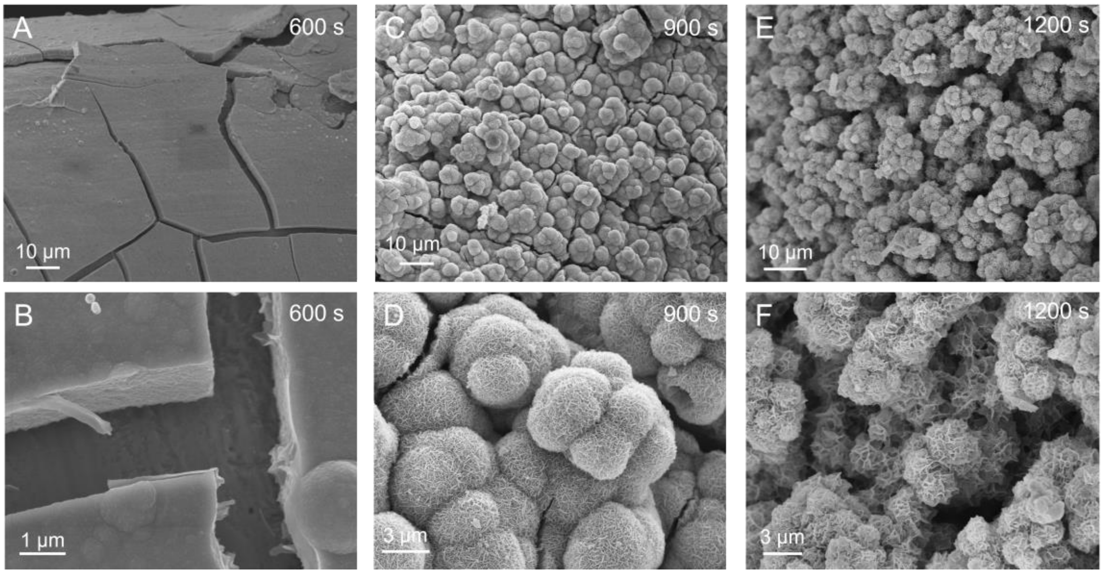

Figure S4. SEM images of different samples obtained at various electroplating times: (A, B) 600 s, (C, D) 900 s, and (E, F) 1200 s.

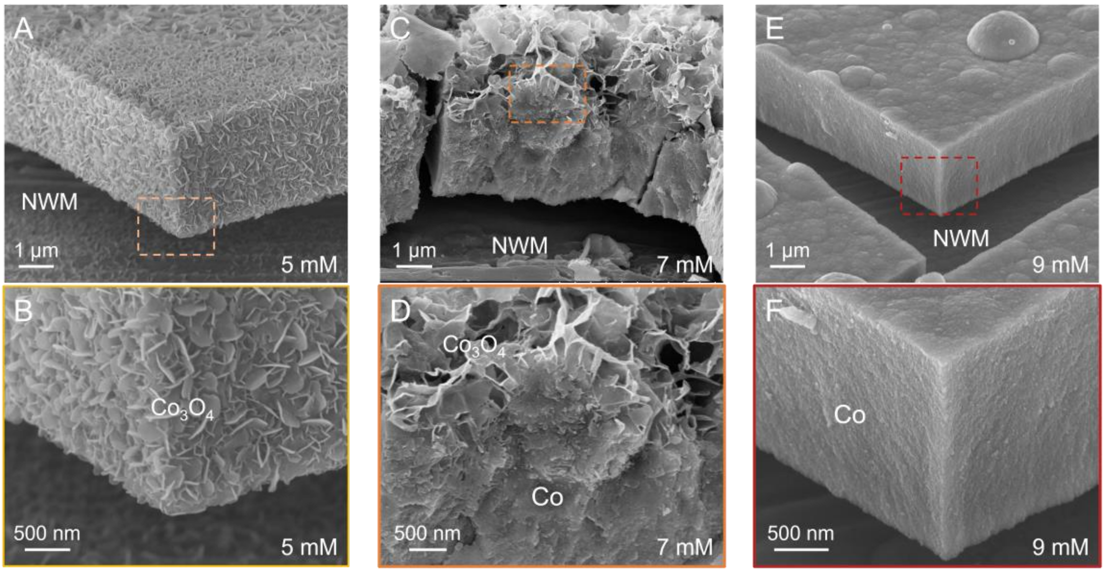

Figure S5. SEM images of different samples obtained at various Co ion concentrations: (A, B) 5 mM, (C, D) 7 mM, and (E, F) 9 mM.

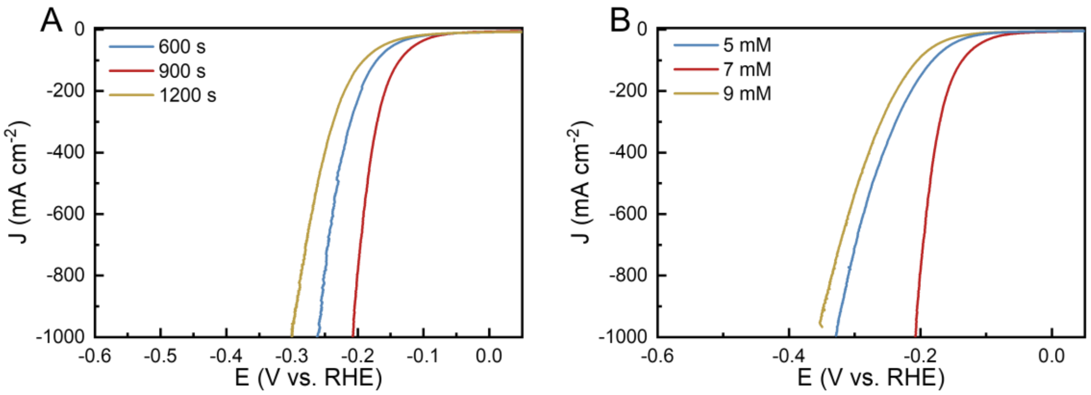

Figure S6. Comparison on HER activity for different electrodes obtained with (A) various electroplating times and (B) different Co ion concentrations.

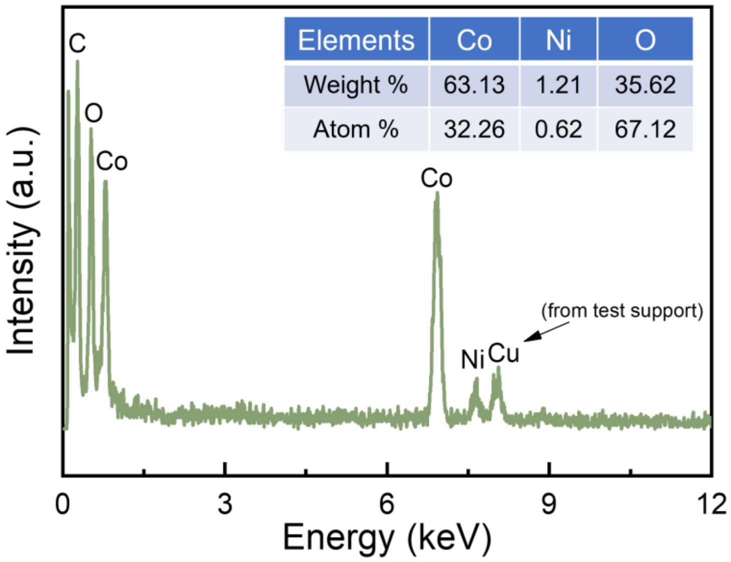

Figure S7. EDS spectrum of Co_3O_4 nanosheets detached from Co_3O_4 electrode.

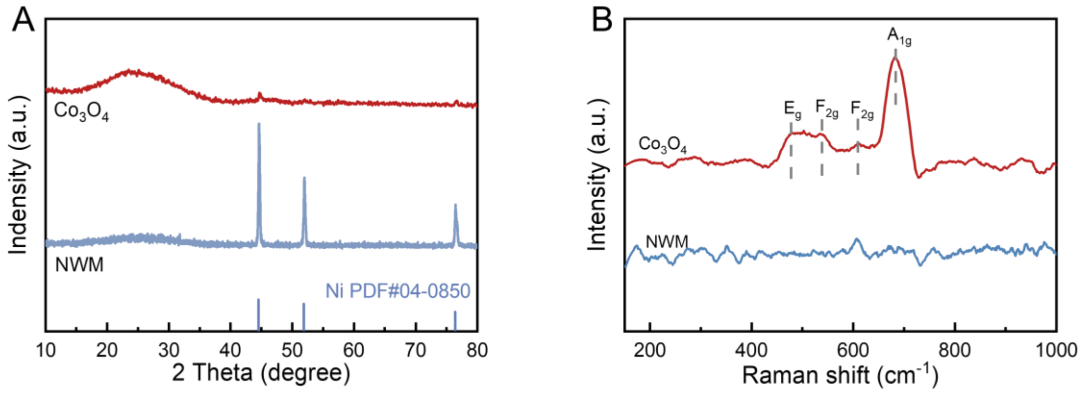

Figure S8. (A) XRD patterns and (B) Raman spectra of Co_3O_4 and pristine NWM electrodes.

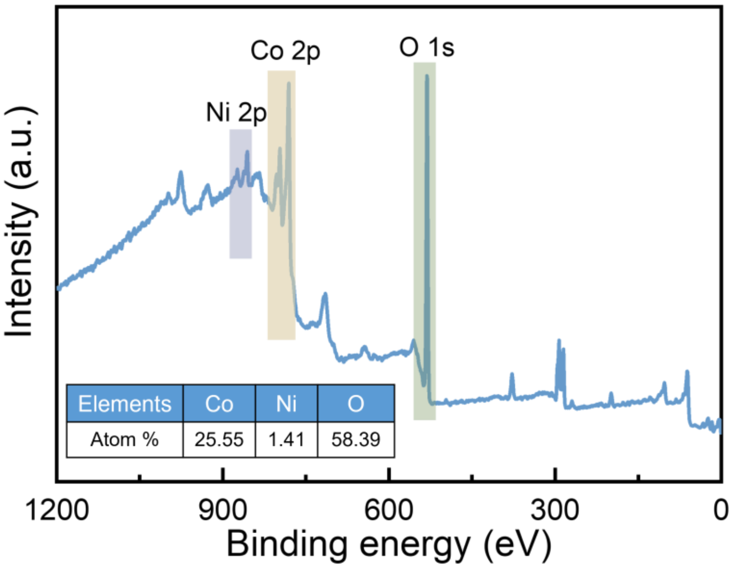

Figure S9. XPS survey spectrum of Co_3O_4 electrode.

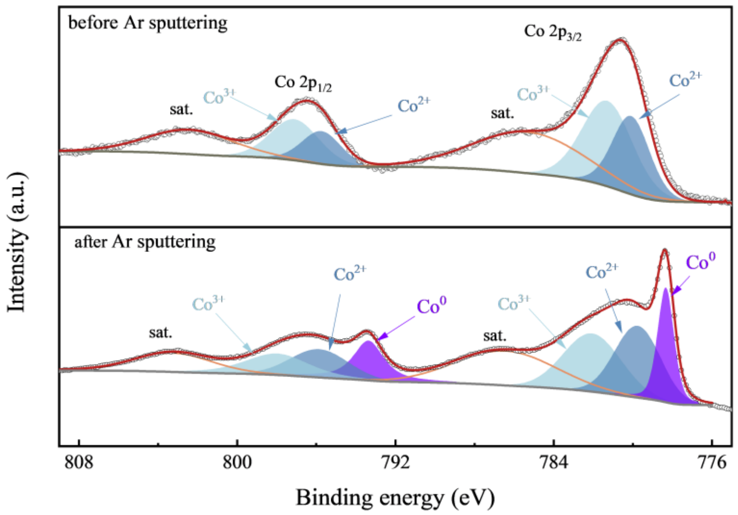

Figure S10. High-resolution Co-2p XPS spectra of Co_3O_4 electrode before and after Ar sputtering.

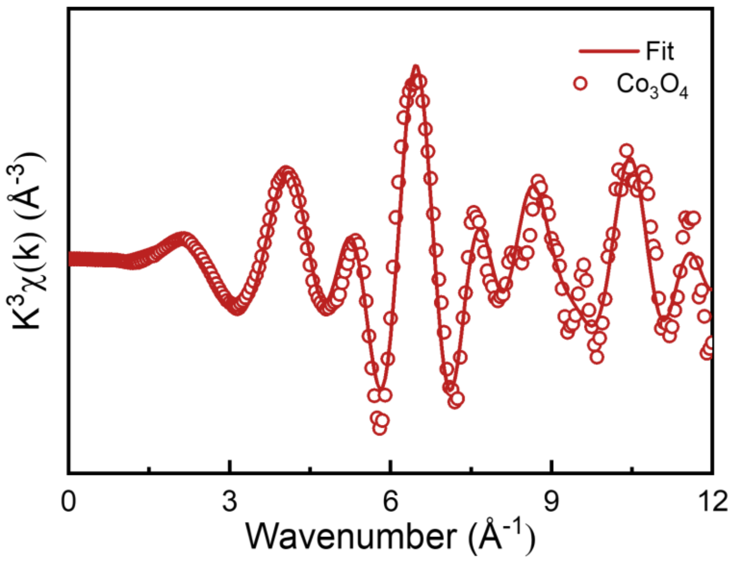

Figure S11. Fitting curve of Co K-edge FT-EXAFS spectrum in *k*-space of Co_3O_4 electrode.

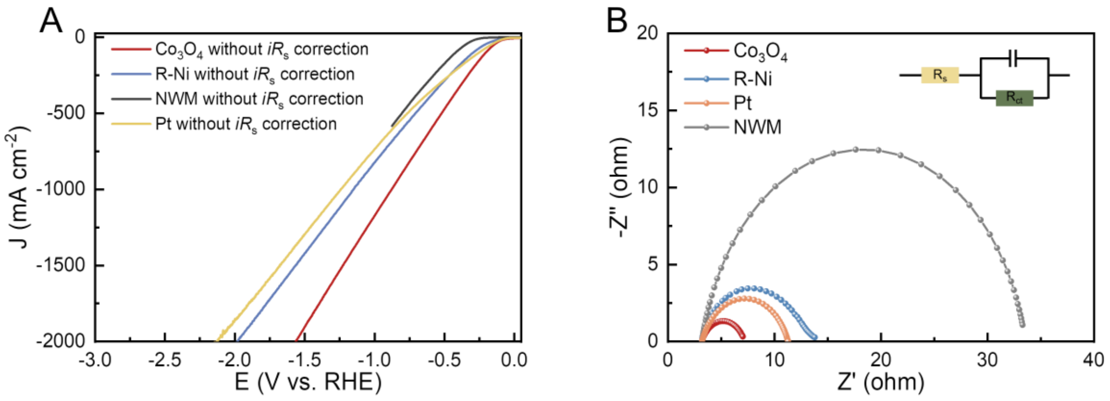

Figure S12. (A) LSV curves without iR_s corrections and (B) EIS spectra of Co_3O_4, R-Ni, NWM, and Pt electrodes. Note: R_s is determined to be 3.2 Ω by EIS spectra.

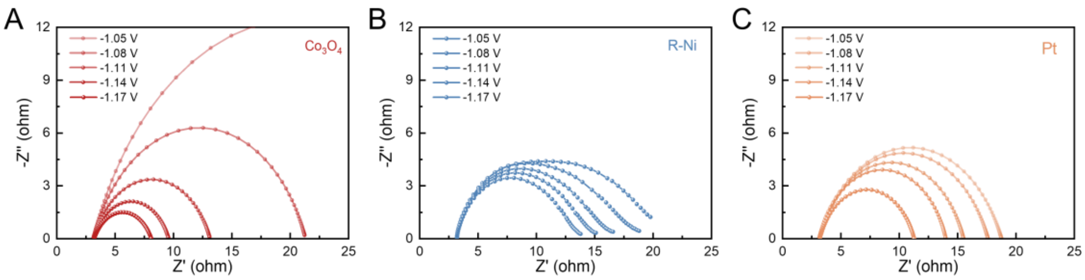

Figure S13. Nyquist plots of operando EIS spectra with varying potentials vs. Hg/HgO for (A) Co_3O_4, (B) R-Ni, and (C) Pt electrodes.

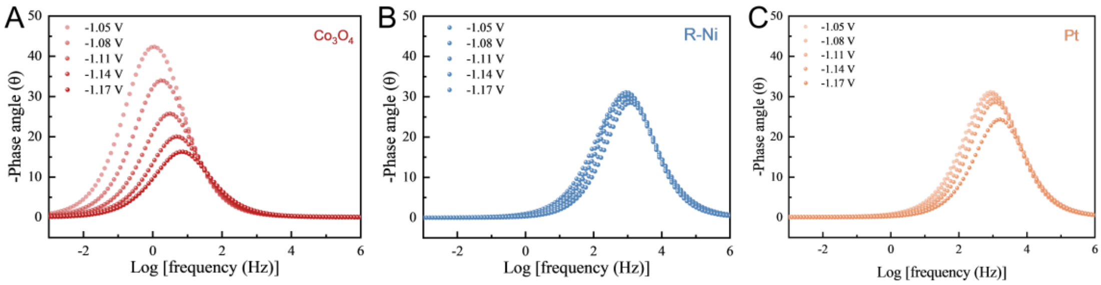

Figure S14. Phase angle vs. log[frequency] plots of operando EIS spectra with varying potentials vs. Hg/HgO for (A) Co_3O_4, (B) R-Ni, and (C) Pt electrodes.

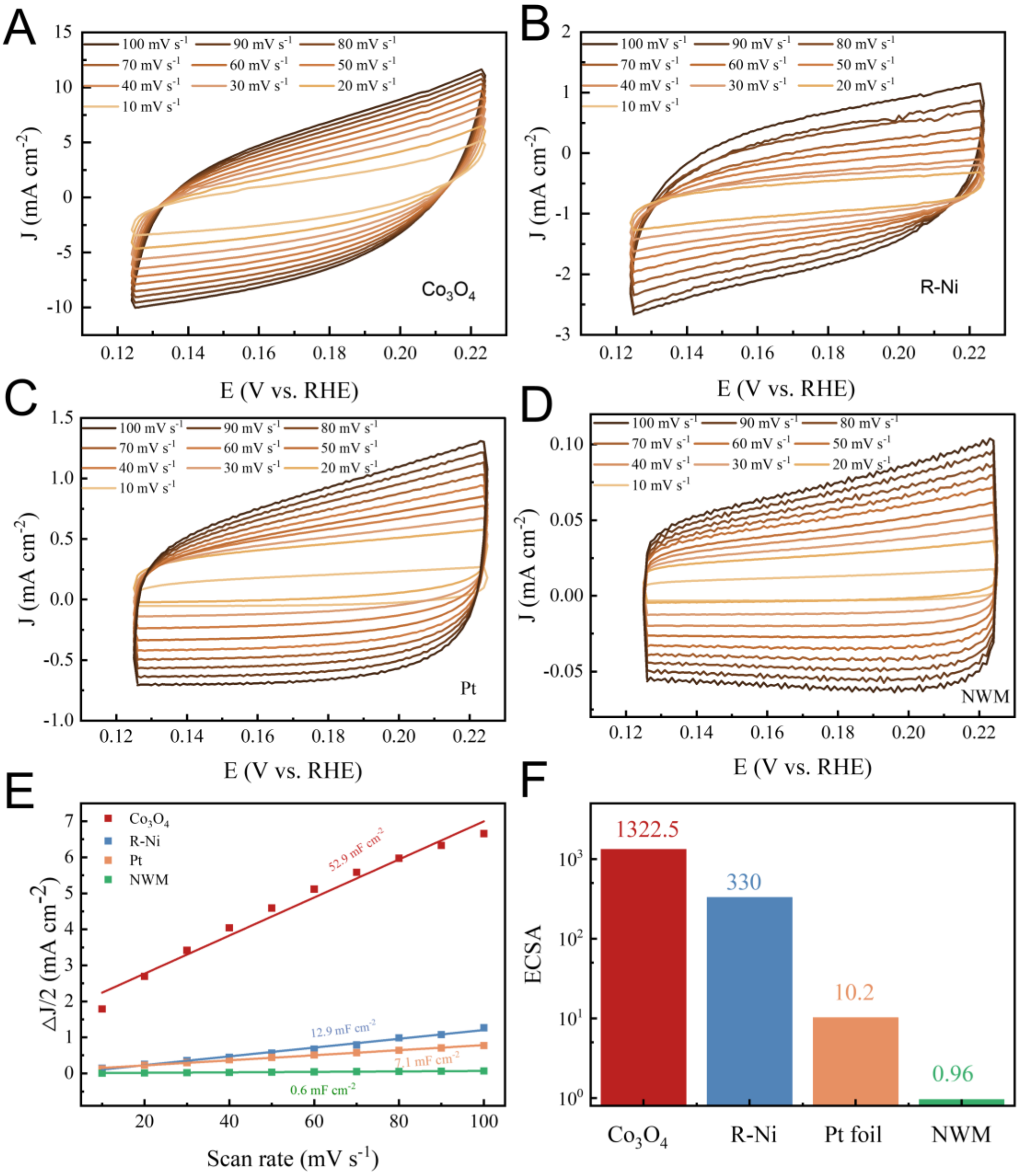

Figure S15. Faraday current-free CV curves of (A) Co_3O_4, (B) R-Ni, (C) Pt, and (D) NWM. (E) Fitting plots of half current difference vs. scan rate to determine C_{dl} values, and (F) the corresponding ECSA values.

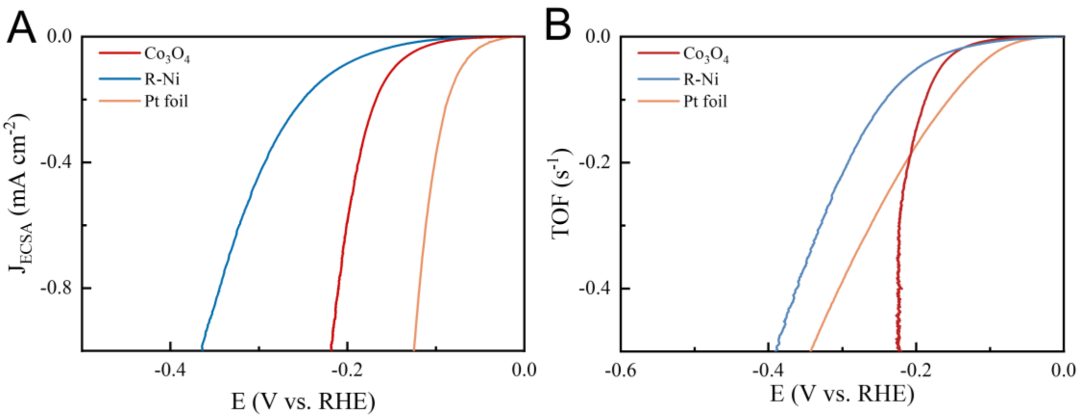

Figure S16. (A) ECSA-normalized LSV curves and (B) TOF curves of Co_3O_4, R-Ni, and Pt foil.

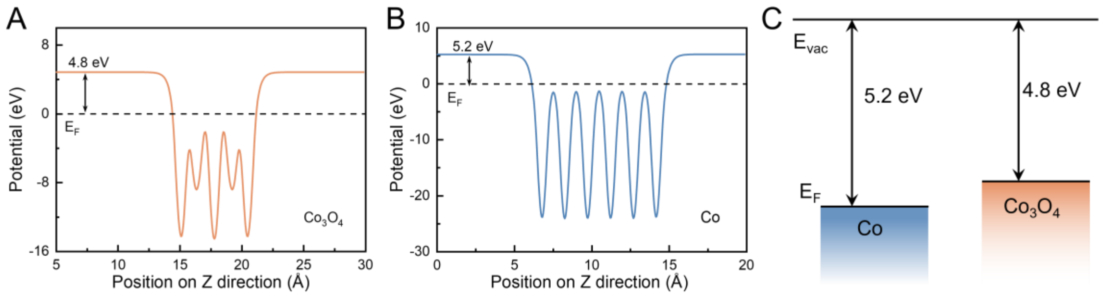

Figure S17. The plane-averaged electrostatic potentials of (A) Co_3O_4 and (B) Co. The Fermi levels are set to zero. (C) The relative arrangement of Fermi levels on each side before electronical interaction between Co and Co_3O_4.

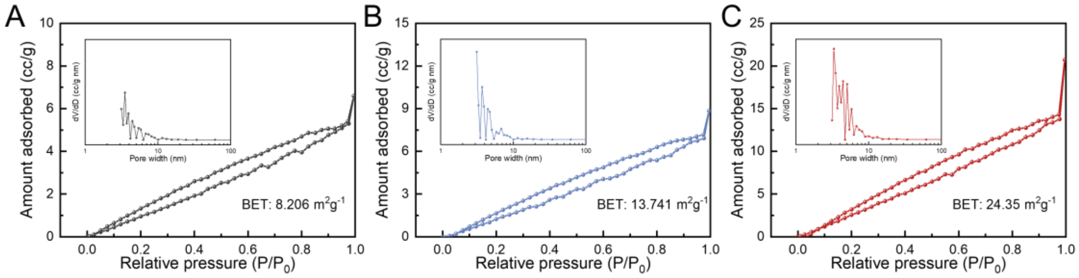

Figure S18. Nitrogen adsorption-desorption isotherms of (A) NWM, (B) R-Ni, and (C) Co_3O_4 electrodes, with the analysis on BET specific surface areas and pore diameters (Insets).

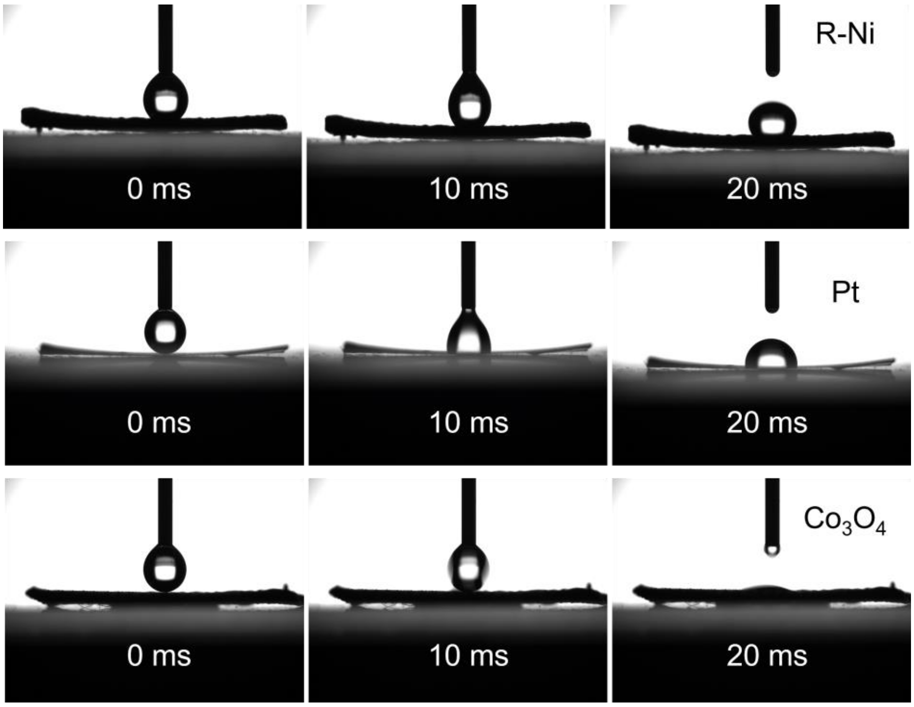

Figure S19. Dynamic droplet wetting tests for R-Ni, Pt, and Co_3O_4 electrodes.

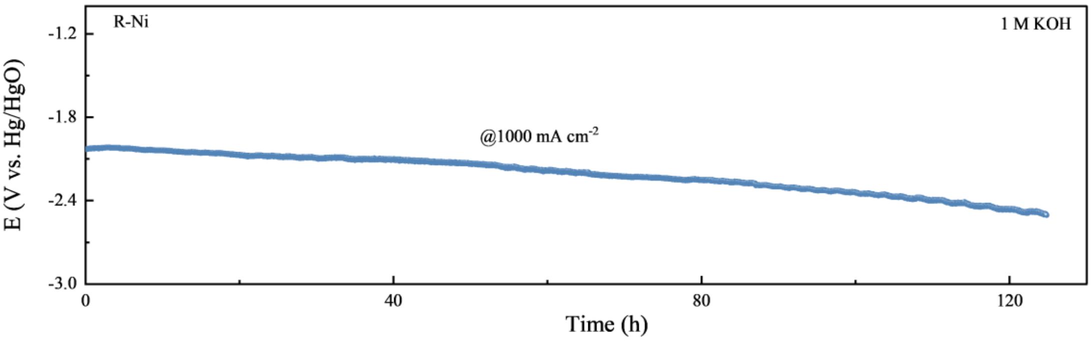

Figure S20. CP stability test for commercial R-Ni electrode at 1000 mA cm^{-2}.

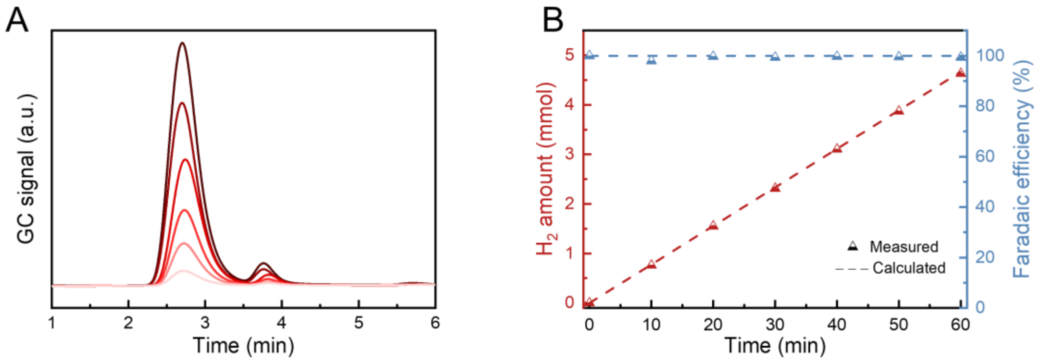

Figure S21. (A) Time-dependent gas chromatography (GC) signals for H_2 and O_2 evolutions during electrolysis at 1000 mA cm^{-2}. (B) Time-dependent H_2 amount and the corresponding Faraday efficiencies.

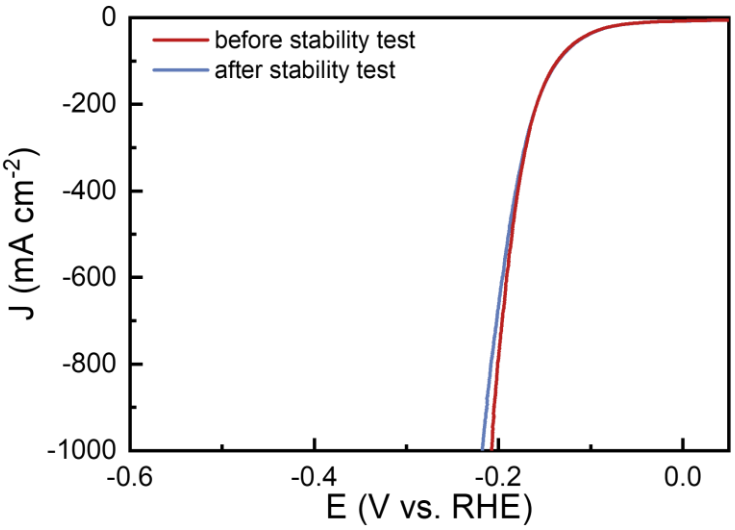

Figure S22. LSV curves of Co_3O_4 electrode before and after stability test.

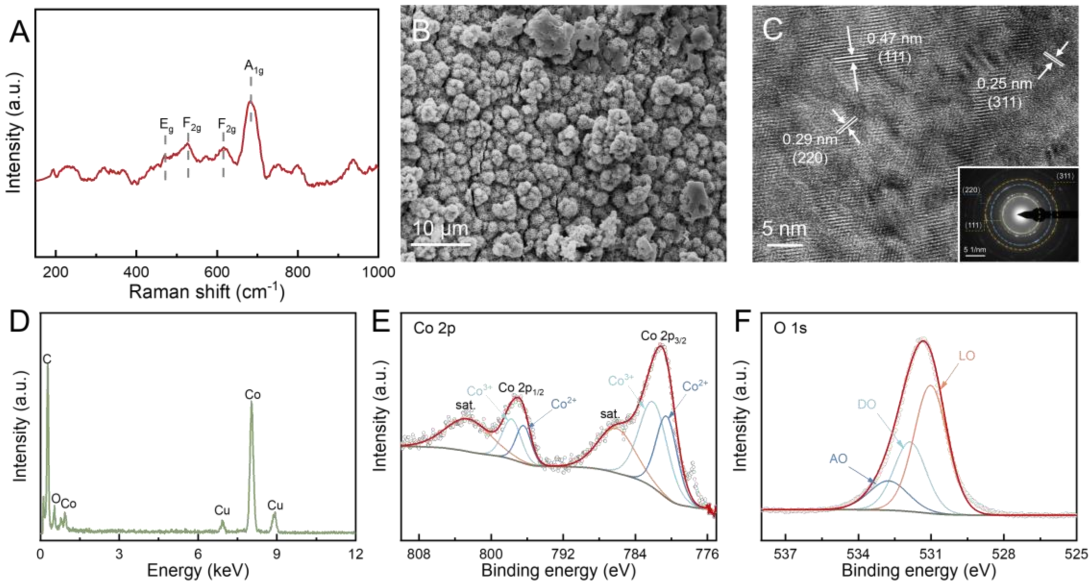

Figure S23. (A) Raman spectrum, (B) SEM image, (C) HRTEM image, (D) EDS spectrum, XPS spectra at (E) Co-2p and (F) O-1s cores of Co_3O_4 electrode after stability test.

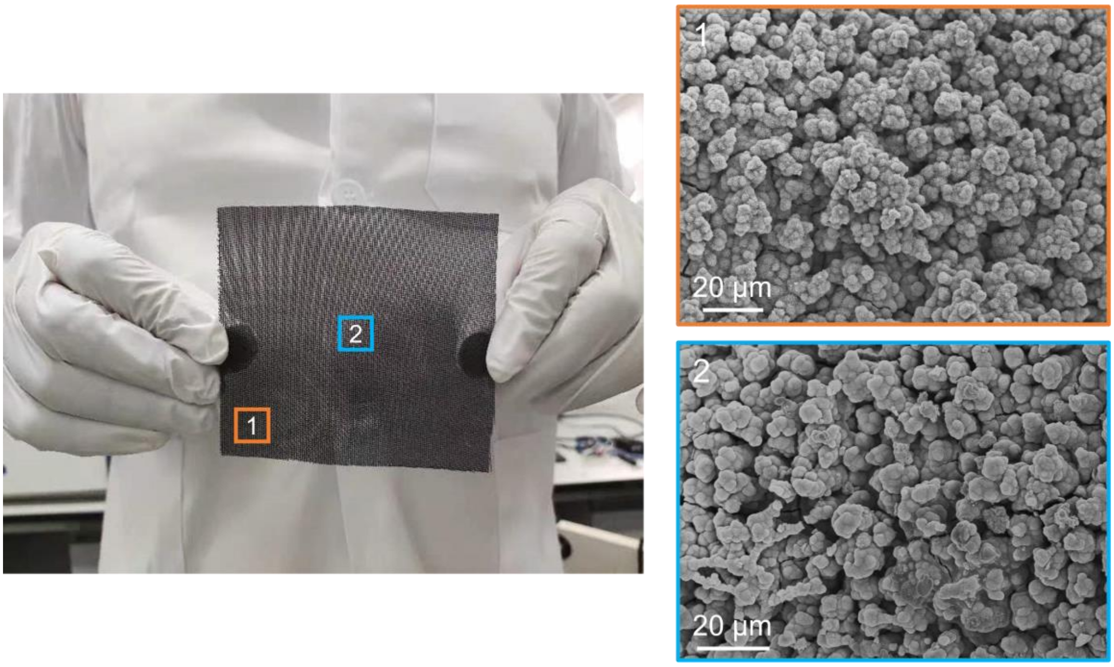

Figure S24. Photograph of a piece of Co_3O_4 electrode of 10 cm × 10 cm in size, and SEM images in two different regions.

Table S1. Mass values at various treatment times (M_x, x denotes x min) during the ultrasonic destruction tests for Co_3O_4, R-Ni, and NWM electrodes.

Sample	M_0 (mg)	M_{10} (mg)	M_{20} (mg)	M_{30} (mg)	M_{40} (mg)	M_{50} (mg)	M_{60} (mg)
Co_3O_4@NWM	174.9	174.1	173.5	172.5	172.1	171.6	171.2
R-Ni@NWM	189.2	188.2	184.9	181.4	177.9	173.5	169.4
NWM	165.0	165.0	165.0	165.0	165.0	165.0	165.0

Table S2. Structural parameters of Co_3O_4 from the Co K-edge EXAFS fitting.

Sample	Shell	CN	R (Å)	σ^2 (10^{-2}Å^2)	R-factor (%)	E_0 (eV)	ΔE_0 (eV)
Co_3O_4	Co-O	3.38±0.28	1.92±0.02	0.2±0.001	0.01	7727	-4.67±0.82
	Co_{otc}-Co_{otc}	6.55±1.36	2.87±0.02	1±0.002			
	Co_{tet}-Co_{tet}	3.67±1.36	3.34±0.02	0.5±0.003			

Note: The background subtraction, merging, normalization, and fitting of the XAS data were performed by Demeter software package. CN is the coordination number for the absorber-backscatterer pair, R is the average absorber-backscatterer distance, σ^2 is the Debye-Waller factor. The data range used for data fitting in k-space (Δk) and R-space (ΔR) are 2.0-12.0 Å^{-1} and 1.0-1.3 Å, respectively.

Table S3. Comparison on overpotentials at typical current densities (500, 1000 and 2000 mA cm^{-2}) for Co_3O_4 with advanced HER catalysts reported in literature.

Catalysts	Support	Electrolyte	η@500 mA cm^{-2} (mV)	η@1000 mA cm^{-2} (mV)	η@2000 mA cm^{-2} (mV)	Reference
Co_3O_4	NWM	1 M KOH	185	207	225	*This work*
$CuMo_6S_8$	Cu foam	1 M KOH	290	320	328	*Nat. Commun. 2022, 13, 6382*
Ni/MoO_2	Carbon nanotube	1 M KOH	220	267	--	*Nano-Micro Lett. 2022, 14, 20*
Ni_2P	Ni foam	1 M KOH	242	306	417	*J. Am. Chem. Soc. 2019, 141, 7537*
Nano-KFO	Ni foam	1 M KOH	281	303	343	*J. Mater. Chem.A 2021, 9, 7586*
a-$MoWS_x$/N-RGO	Graphite rod	1 M KOH	275	348	--	*Adv. Sci. 2022, 9, 2202445*
NMFSOH	Ni foam	1 M KOH	173	200	231	*Adv. Energy Mater. 2023, 2301222*
P-NiMoHZ	Ni foam	1 M KOH	170	210	--	*Nat. Commun. 2021, 12, 5960*
NiFeLDH	Ni foam	1 M KOH	356	--	--	*Small 2021, 18, 2104354*
MoS/MoC	Ti foil	1 M KOH	191	220	--	*Nat. Commun. 2019, 10, 269*
Ni/Y_2O_3	Graphite rod	1 M KOH	170	230	--	*Adv. Energy Mater. 2024, 14, 2303563*
MnO-CoP	Ni foam	1 M KOH	187	260	--	*Nano Lett. 2023, 23, 9087−9095*
$Ni_{0.96}Co_{0.04}P$	Ni foam	1 M KOH	161	251	--	*Adv. Funct. Mater. 2023, 33, 2205161*
N-P-B	Carbon paper	1 M KOH	276	345	--	*Energy Environ. Sci. 2020, 13, 102–110*

Table S4. Price of the scale-up fabrication of Co_3O_4 electrode in comparison with commercial Raney® Ni electrode.

Electrode	Content item	Supplier	Price (US$)	Usage (m^{-2})	Cost (US$ m^{-2})	Price (US$ m^{-2})
Co_3O_4@NWM (This work; loading 100 g m^{-2})	Cobalt acetate tetrahydrate	China National Pharmaceutical Group Chemical Reagent Co., Ltd	130 kg^{-1}	248 g	32.24	45.0
	Ammonium chloride	China National Pharmaceutical Group Chemical Reagent Co., Ltd	5 kg^{-1}	1070 g	5.35	
	Thiourea	China National Pharmaceutical Group Chemical Reagent Co., Ltd	5.3 kg^{-1}	76 g	0.41	
	Nickel wire mesh	Jiangsu Green Hydrogen Electrode Co., Ltd	5.0 m^{-2}	1 m^2	5.0	
	Electroplating process		2.0 m^{-2}	15 min	2.0	
Raney® Ni@NWM (Commercial; loading 240 g m^{-2})	Raney® Ni	Jiangsu Leini Metal Technology Co., Ltd	110 kg^{-1}	240 g (loading) 300 g (wasting)	59.4	72.4
	Nickel wire mesh	Jiangsu Green Hydrogen Electrode Co., Ltd	5.0 m^{-2}	1 m^2	5.0	
	Electroplating process & Alkali activation		8.0 m^{-2}	1 m^2	8.0	

Video S1. Video of water splitting at 1000 mA cm^{-2} in an industrial electrolysis system.
